# Study on the Bending and Shear Behavior of a New Type of Wet Joint in Precast Concrete Deck for Composite Bridges

**DOI:** 10.3390/ma17246252

**Published:** 2024-12-21

**Authors:** Yan Wang, Long Hu, Wei Li, Guangshuai Zhang, Bin Han, Jin Di, Peng Fei, Xiaofeng Duan, Jinying Dong, Fengjiang Qin

**Affiliations:** 1 Shandong Expressway Qingdao Construction Management Co., Ltd., Qingdao 266300, China; wangyan471564008@163.com (Y.W.); liwei383768366@163.com (W.L.); 2 Key Laboratory of New Technology for Construction of Cities in Mountain Area, School of Civil Engineering, Chongqing University, Chongqing 400000, China; hanbin202404@163.com (B.H.); dijin@cqu.edu.cn (J.D.); 3 Shandong Expressway Engineering Construction Group Co., Ltd., Jinan 250102, China; zhangguangshuai10@163.com (G.Z.); feipeng952265607@163.com (P.F.); duanxiaofeng1273@163.com (X.D.); dongjinying1098@163.com (J.D.); 4 China Construction Third Engineering Bureau Co., Ltd., Wuhan 430073, China

**Keywords:** bridge engineering, wet joint, experimental and finite element research, u-bars, headed bars, combined bending-shear load

## Abstract

According to the mechanical characteristics of joints in steel–concrete composite bridge decks under the combined bending and shear, improved joint details with simple structure and convenient construction were studied, including lapped U-bars, lapped headed bars, and lapped hook bars. In order to test the mechanical properties of the three joint details and compare them with the existing lapped/welded linear bars, the tests of five specimens were carried out. The cracking load, ultimate load, failure mode, crack pattern, and reinforcement strain were analyzed. The test results showed that the joint with lapped U-bars and hook bars exhibited ductile failure, while the joint with lapped headed and lapped/welded linear bars exhibited brittle failure. The cracking load of the five specimens was basically the same. The crack first occurred at the interface of pre-cast concrete and wet joints. When the ultimate bearing capacity was reached, the vertical main cracks were generated near the interface of the lapped U-bars, lapped hook bars, and welded linear bars specimens. The diagonal cracks were generated at the wet joint of the lapped headed bars specimen and lapped linear bars specimen. The lapped U-bars specimen had the highest bearing capacity, which was 22.8%, 14.2%, 50.4%, and 32.1% higher than the capacities of the lapped headed bars, lapped hook bars, lapped linear bars, and welded linear bars specimens, respectively. The load–deflection curves and crack mode obtained from the FEM of the lapped U-bars joint specimen were consistent with the test results. The bearing capacity of the FEM (351.3 KN) was 1.8% less than the test result (357.6 KN), which indicates that the bearing capacity calculated by the finite element model is reliable. There are 80 models with varying lap lengths and concrete strengths. The self-organizing migrating algorithm was used to fit the coupling effect of lap length and concrete strength. Based on doubly reinforced beam flexural capacity formulas, a bearing capacity calculation for lapped U-bars joint was proposed. The mean value of the formula calculation result and the finite element result ratio is 1.03, and the variance is 0.0004.

## 1. Introduction

In steel–concrete composite bridges, wet joints are commonly used to connect prefabricated bridge decks. These wet joints are designed to transmit the combined bending and shear forces acting on the bridge decks. At present, scholars at home and abroad have conducted extensive research on the mechanical properties of U-bar lapping, head bar lapping, and linear bar lapping under the conditions of pure tension, pure bending, and tension bending.

Hao [1] conducted 36 pure tension tests and 19 pure bending tests on wet joints with U-bars, with all specimens designed using a 1-to-2 staggered lap joint configuration, where each U-bar is staggered and overlapped with two others in a pairwise manner. Ong et al. [2] proposed a strut-and-tie model for calculating the ultimate tensile strength of wet joints with U-shaped bars. In order to propose a wet joint configuration suitable for the negative moment zone of prefabricated bridge decks in steel–concrete composite bridges, Gordon [3,4] conducted 18 pure tension tests on wet joints reinforced with U-bars. The U-bars were arranged in a staggered configuration, and the specimens were categorized into two groups: those with 3–4 symmetrical lap joints and those with 3–3 asymmetrical lap joints. The experimental results demonstrated that when the transverse reinforcement ratio in the core concrete was adequate, the strength of the wet joints could be predicted with relatively high accuracy based on the strength of the U-bars. Ryu et al. [5] conducted nine pure bending tests on wet joints reinforced with U-bars, featuring a 5–5 staggered lap joint configuration for the U-bars. They discovered that the overlapping portion within the loop joint exhibits comparable or even superior mechanical properties to those of an RC beam section devoid of joints. However, it is crucial to acknowledge the potential for a decrease in stiffness and strength at the casting interface area of RC members featuring loop joints, particularly when the joint widths are inadequately narrow. Li et al. [6] performed seven pure bending tests on bridge decks, with wet joints incorporating either lap-spliced headed bars or welded linear bars, along with a cast-in-situ specimen serving as a control group. Based on the test results, the article recommends adopting wet joints with lap-spliced headed steel bars using a lap length of 152 mm as an alternative to the existing wet joints with welded linear bars. The 152 mm headed bar detail can provide a continuous force transfer in the longitudinal joint and minimize the width of the joint to accelerate bridge construction. To enhance the speed of bridge construction, Ma et al. [7] investigated the use of tightly bent U-bars in the transverse wet joints of prefabricated bridge decks. Their experimental study revealed that U-bars exhibit superior mechanical properties compared to headed bars. Additionally, the study examined the impact of concrete strength, steel bar diameter, and lap length on the performance of wet joints reinforced with U-bars through a series of tests. Ma et al. [8] conducted four pure tension tests and four pure bending tests to investigate the impact of steel connection forms. The specimens did not include post-cast wet joints, instead featuring a 3–2 staggered lap joint configuration for the U-bars, with two transverse steel bars with anchor plates at the ends placed within the core concrete.

In another study, Ma et al. [9] examined wet joints reinforced with tightly bent U-bars under pure bending loads and utilized a strut-and-tie model to analyze the force distribution within the wet joints. Based on their findings, design recommendations were provided for the spacing of U-bars, lap length, diameter of transverse steel bars, and concrete strength of the wet joints. They recommend the use of U-bars since the U-bar detail created a less-congested joint, which made it the easiest to construct. Joergensen and Hoang [10] conducted pure tension tests on wet joints reinforced with U-bars, revealing that the strength of these wet joints is influenced by factors such as the lap length of the U-bars, the spacing between U-bars, and the transverse reinforcement ratio of the core concrete. Joergensen [11] performed tests on wet joints reinforced with U-bars under combined bending and tension loads, proposing a plastic calculation model for predicting the strength of such wet joints under these combined loads. Furthermore, Joergensen and Hoang [12] delved deeper into the strength of wet joints reinforced with U-bars under combined bending and tension, presenting plastic calculation models tailored to different failure modes. Zhu Yu and Ding Dehao [13] pointed out that the tensile force in the longitudinal steel reinforcement of U-bar wet joints is balanced not by the bond between steel and concrete but by the shear resistance provided by the core concrete tenons. Based on this understanding, they established a calculation formula for the lap length of U-bars. In a separate study, Li and Jiang [14] investigated the use of headed bars in wet joints between prefabricated T-beams to expedite bridge construction. Their experimental design included five specimens with headed bar connectors and one cast-in-situ control specimen. Based on the test results, they recommended the adoption of headed bars with a lap length of 152 mm for wet joints under pure bending and combined bending-shear loads. However, the details of their proposed bulk rebar only apply to wet joints with small widths.

Arafa et al. [15] conducted a study on the use of ultra-high-performance fiber-reinforced concrete (UHPFRC) wet joints for connecting prefabricated bridge decks, with reinforcement provided by glass fiber-reinforced materials. Their experimental design included eight specimens, three of which served as control specimens. The tests employed cantilever loading to simulate the bending moments and shear forces experienced by wet joints in negative moment zones. The experimental results show that the wet joints with GFRP bars have a higher cracking load, the UHPFRC joints showed adequate strength and performance, and the failure occurred in the spliced concrete panels. In another study, Vella et al. [16] examined the performance of wet joints with headed bars connectors under pure bending loads. Their experimental investigation focused on the impact of concrete strength, out-of-plane displacement of prefabricated panels, and shear studs on the strength, ductility, and crack width of the wet joints. It is found that in order to realize the ductile failure of the wet joints of large-head rebar, the lap length and concrete strength must meet specific conditions. Vella et al. [17] investigated the mechanical properties of narrow wet joints using large-headed steel connectors through pure tension tests, with a focus on understanding the behavior of wet joints in tension zones. According to the experiment, they found that the lap strength is limited by headed bar strength, transverse bar yielding, or concrete failure. Su Qingtian et al. [18], in response to the stress characteristics at wet joints in prefabricated deck systems for composite bridges and the existing reinforcement connection forms, proposed a novel curved reinforcement connection that is structurally simple and facilitates easy construction. Full-scale models were subjected to axial tension tests, and the mechanical performance of this curved reinforcement connection was compared with existing straight reinforcement and U-bars. The results indicated that the curved reinforcement connection is feasible and reliable for practical engineering applications.

Zhang Yang and Chen Bei [19] conducted a study on wet joints reinforced with U-bars in ultra-high-performance concrete (UHPC). Their findings revealed that the bending and tensile resistance of joint panels with different wet joint configurations surpassed that of intact bridge decks. Specifically, joint panels with drilled holes and densely arranged reinforcement exhibited significantly enhanced bending and tensile resistance. Wedge-shaped and diamond-shaped joint configurations followed suit, while rectangular joint configurations performed the least effectively. Zhang Yongtao and Tian Fei [20] designed and fabricated two bridge deck specimens with U-bar wet joints and one control specimen. The experimental results demonstrated that wet joints did not compromise the bending capacity of the bridge deck, and ultra-high-performance concrete (UHPC) significantly enhanced the crack resistance of the wet joints. Qi et al. [21,22] investigated the bending performance of a novel UHPC wet joint, with test parameters including interface treatment methods between old and new concrete, wet joint materials, reinforcement lap configurations, and prestressing levels. Di et al. [23] conducted tests on four specimens with U-bar wet joints under combined bending and shear loads, examining parameters such as U-bar connection types and wet joint shapes. Based on experimental and finite element analysis results, they recommended the adoption of T-shaped wet joints with U-bar lap connections. Qiu et al. [24] conducted a study on the flexural performance of wet joints in 12 prefabricated ultra-high-performance concrete (UHPC) panels. The experimental parameters included joint connection shapes (T-shaped and rectangular), steel bar diameters, restraint conditions of the prefabricated UHPC panels, and roughness of the joint cross-sections. The findings revealed that rectangular joints exhibited lower crack resistance, stiffness, and flexural capacity after cracking yet demonstrated higher ductility. Conversely, T-shaped joints showed higher crack resistance, stiffness, and flexural capacity but lower ductility. Additionally, increasing the interface roughness of the prefabricated UHPC panels was found to enhance their crack resistance with minimal impact on other mechanical properties. Han et al. [25] conducted pure bending tests on six types of wet joint configurations. The test specimens were evaluated based on their bearing capacity, failure modes, crack control, stiffness, and steel strain. They found that U-shaped steel bars exhibited higher bearing capacity and ductile failure characteristics. In contrast, headed bars and linear bars demonstrated lower bearing capacity and showed brittle failure modes. Among the configurations tested, the lapped U-bars with T-shaped joint detail were found to be suitable for joint fabrication between prefabricated concrete panels due to their favorable performance. Jia et al. [26] designed four prefabricated bridge decks with wet joints and one monolithic cast-in-situ bridge deck. Axial tensile loading tests were conducted to evaluate the tensile performance of these five bridge deck panels. Based on the strut-and-tie model and failure mode, a modified calculation formula was proposed for estimating the tensile capacity of precast bridge deck panels. Ren et al. [27] investigated the shear performance of precast bridge deck panels with UHPC wet joints, a total of seven bridge deck panels (BDPs) were designed and conducted by four-point bending tests. The shear capacity formulas in the design codes were verified in comparison with the test results. The deviation of theoretically calculated values from test results was within 10%. Huang et al. [28] designed a total of nine specimens incorporating three different U-bar joint details and examined them through four-point bending tests. The experimental results indicate that welded splices can be omitted without compromising the moment capacity of wet joints. Additionally, 182 finite element models were established to investigate the effects of lap length, concrete and steel grades, and stirrups on the flexural performance of U-bar joints. Consequently, the minimum lap length for splicing U-bars in double-notch lap splices was determined, and a calculation formula was proposed to estimate this minimum lap length with reasonable accuracy. Xiao et al. [29] conducted full-scale tests of three steel-UHPC composite slabs with dovetail-shaped wet joints or newly proposed construction-friendly rectangular wet joints. Based on the comprehensive analysis method combining the bond-slip method and the non-slip method, a crack width prediction model was obtained. The modified model can give satisfactory prediction results of crack width for both interfacial and non-interfacial cracks.

A comprehensive review of current research reveals that scholars from both domestic and international circles have conducted extensive studies on the mechanical performance of wet joints with various configurations, including U-bars lap splice wet joints [1,2,3,4,5,7,8,9,10,11,12,13,19,21,22,23,25,26,27,28], large-headed bars lap splice wet joints [6,7,14,16,17,25], and straight bars lap splice wet joints [15,24,25,26,27,29]. However, a comparative analysis of the mechanical properties of these different wet joint configurations remains scant in the literature. Existing research primarily focuses on the mechanical behavior of wet joints under pure tension, pure bending, and combined bending-tension loads, with a notable lack of studies on the mechanical performance of wet joints in steel–concrete composite bridge decks under combined bending-shear loads.

To address this gap and facilitate a comparison of the mechanical properties of different wet joint configurations under combined bending-shear actions, as well as to propose a suitable wet joint configuration for steel–concrete composite bridges, this paper presents five wet joint specimens: lapped U-bars joints (LU), lapped headed bars joints (LH), lapped hooked bars joints (LO), lapped linear bars joints (LL), and welded linear bars joints (WL). The test subjects the specimens to combined bending-shear loads, comparing their bearing capacity, failure modes, crack distribution, failure deflection, and reinforcing steel bar strain. Additionally, a nonlinear finite element analysis is conducted on the U-bars lap splice wet joint specimens. Based on the experimental results and parametric analysis of 80 finite element models, a formula for calculating the bearing capacity of U-bars lap wet joints is proposed.

## 2. Experimental Scheme

### 2.1. Specimen Details

**Figure 1:**
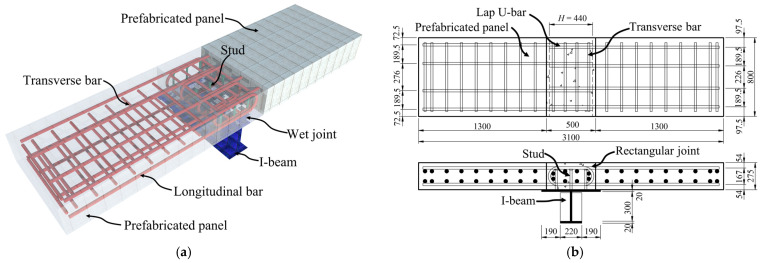

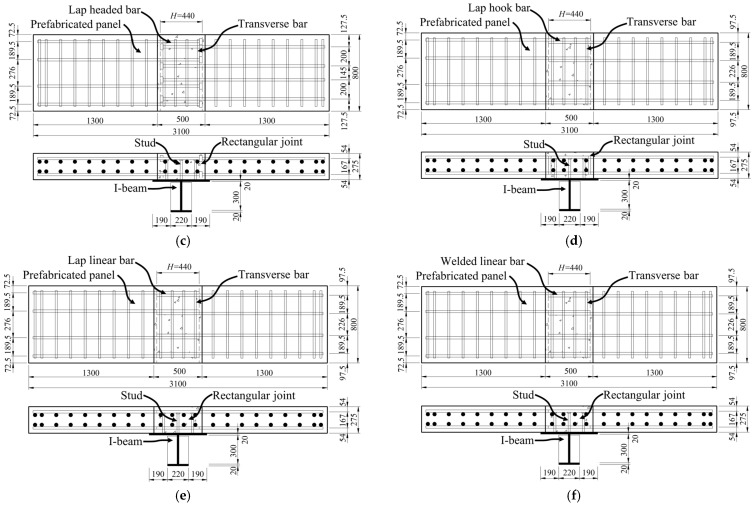
Dimensions of specimens (unit: mm). (**a**) Three-dimensional view of typical specimens; (**b**) Lapped U-bars joint; (**c**) Lapped headed bars joint; (**d**) Lapped hooked bars joint; (**e**) Lapped linear bars overlap joint; (**f**) Welded linear bars joint.

All specimens are composed of concrete bridge decks and I-shaped steel beams with studs. The two ends of each specimen are prefabricated concrete plates, while the middle wet joint is cast in situ. The bridge deck employs C60 in standard GB 50010 [30] with dimensions of 3100 mm × 800 mm × 275 mm. The longitudinal steel bars within the bridge deck have a diameter of 25 mm, and the transverse steel bars have a diameter of 22 mm. The studs within the wet joint measure Φ22 mm × 200 mm. The I-shaped steel beams are made of Q345 in standard GB 50017 [31], with an upper flange width of 600 mm, a lower flange width of 220 mm, a length of 800 mm, and a height of 340 mm. To prevent buckling of the web of the I-shaped steel beam during loading, two stiffeners that are not welded to the upper flange are specifically installed. The detailed configuration of the specimens is shown in Figure 1a, and the dimensions of the specimens are shown in Figure 1b–f.

**Figure 2:**
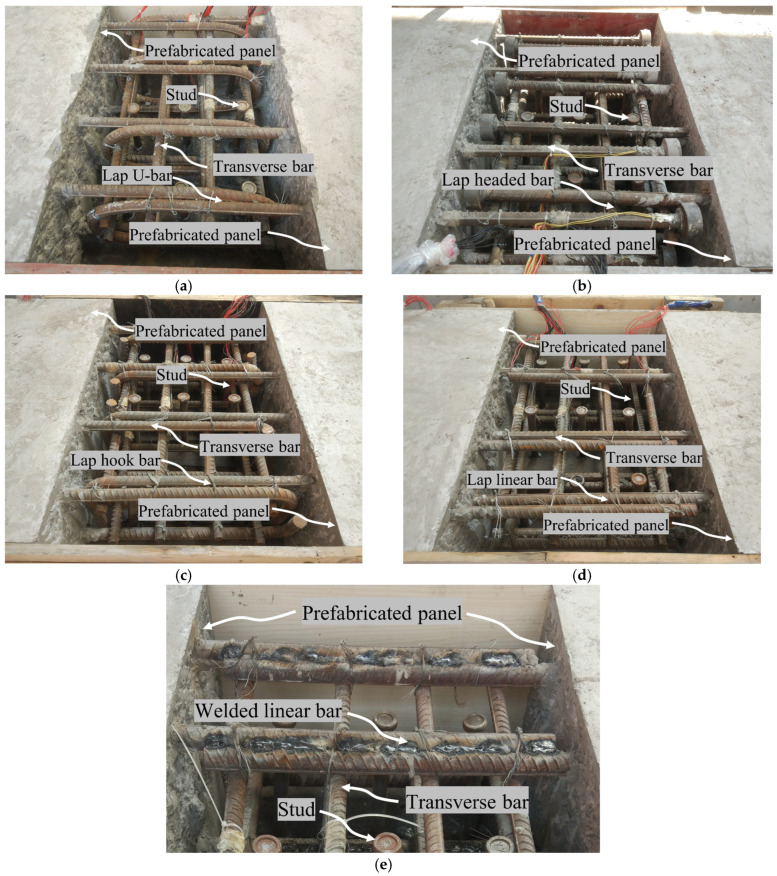
Details of the five joints. (**a**) LU; (**b**) LH; (**c**) LO; (**d**) LL; (**e**) WL.

This research conducted flexural-shear tests on five specimens, which utilized five different connections: lapped U-bars (designated as LU), lapped headed bars (LH), lapped hooked bars (LO), lapped linear bars overlap (LL), and welded linear bars (WL). The wet joint configuration of the specimens is illustrated in Figure 2. All specimens consist of prefabricated panels, transverse reinforcement, connecting reinforcement, studs, and post-cast concrete. Except for the different forms of reinforcement connections, the dimensions and materials of the specimens are identical.

The numbering convention for specimens is as follows: Taking the U-bars specimen numbered LU as an example, the first letter “L” represents the connection method of the steel bar as a lap joint (if the first letter is “W”, it represents welded); the second letter “U” indicates the form of the steel bar as U-bars, with “H”, “O”, and “L” representing headed bars, hooked bars, and linear bars, respectively.

The five specimens studied in this research have a wet joint width of 500 mm and a steel bar lap/welded length of 440 mm. The compressive strength of the wet joint concrete cubes is measured at 62.1 MPa, and the compressive strength of the precast panel concrete cubes is 55.0 MPa. The longitudinal steel bars have a diameter of 25 mm and a yield strength of 460 MPa, while the transverse steel bars have a diameter of 22 mm and a yield strength of 507 MPa. It is worth mentioning that the amount of used longitudinal steel for the lapped linear bar, welded linear bar, and lapped head bar is the same, while the amount of used longitudinal steel for the lapped U-shaped bar and lapped hook bar is slightly higher than others, and the excess amount of used longitudinal steel is not more than 0.5%.

After pouring the precast bridge deck concrete, standard curing was carried out for 28 days. Subsequently, the interface between the wet joint and the precast panel was roughened and wetted before pouring the wet joint concrete. Cubic specimens with a side length of 150 mm were prepared according to the standard GB/T 50081 [32] to determine the concrete strengths of both the precast panels and the wet joints. The tensile tests for the steel bars were conducted in accordance with the standard GB/T 228.1 [33].

### 2.2. Loading Scheme

**Figure 3:**
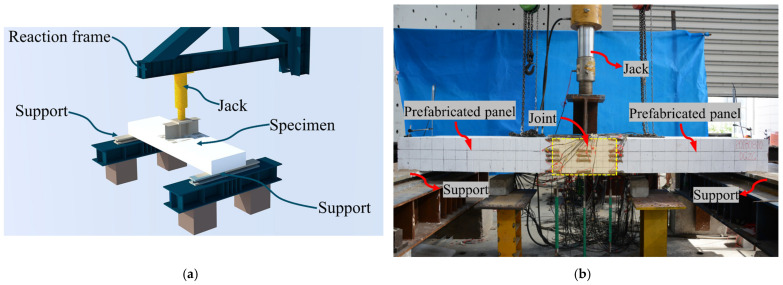
Loading system. (**a**) Loading diagram; (**b**) On-site loading.

All five sets of experiments in this paper utilize a three-point bending-shear loading method, with the loading system configured as shown in Figure 3. The loading device is a 2000 kN hydraulic jack positioned at the mid-span of the specimen. The load is applied to the bottom plate of the I-shaped steel crossbeam through the jack, with a pressure sensor installed between the jack and the crossbeam to accurately measure the applied load. The boundary conditions of the specimen are set as simply supported, with one end being a roller support and the other end being a fixed hinge support. The distance between the two supports is 2900 mm, which is also the calculated span of the specimen. A 50 mm mesh was drawn on the wet joint surface of each specimen, and the mesh size of the remaining surface was 100 mm.

**Figure 4:**
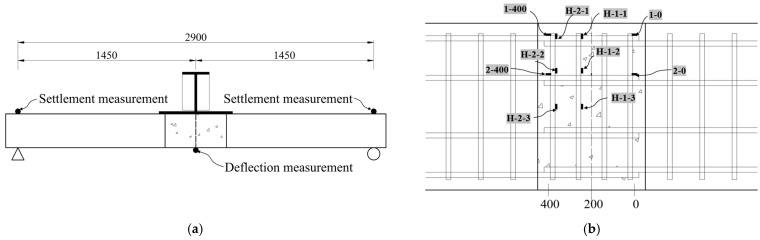
Test-point layout (unit: mm). (**a**) Displacement measurement point layout; (**b**) Reinforcing steel strain measurement point layout.

During the loading process, Linear Variable Differential Transformers (LVDTs) were used to measure the mid-span deflection and bearing settlement. Additionally, the strains of the longitudinal and transverse steel bars at different locations within the wet joint were measured during the test. The arrangement of the LVDTs and strain gauges is shown in Figure 4.

## 3. Test Results and Discussion

### 3.1. Strength of Specimens and Load-Displacement Curves

Through a series of experiments, we obtained specific data on the concrete cracking load (*P_y_*), ultimate load (*P_n_*), and failure deflection (*δ_s_*) of five types of wet joint specimens, with detailed results presented in Table 1. As evident from Table 1, the cracking loads for five types of wet joints can be observed. Specifically, the cracking loads for the lapped U-bar specimens, the lapped headed bar specimens, and the lapped hooked bar specimens are all 40 kN. In contrast, the cracking load for the lapped linear specimens is 38 kN, and that for the welded linear bar specimens is 35 kN. This indicates that the use of lapped/welded linear bar joints results in a reduction in the cracking load of the specimens. Notably, the lapped U-bar specimen exhibited the highest ultimate load, reaching 357.6 kN, which is 22.8%, 14.2%, 50.4%, and 32.1% higher than that of the lapped headed bar specimen (291.2 kN), lapped hooked bar specimen (313.0 kN), lapped linear bar specimen (237.7 kN), and weld linear bar specimen (270.7 kN), respectively. This result fully demonstrates that, compared to headed bars, hooked bars, and linear bars, lapped U-bar joints have significant advantages in providing bearing capacity, this is because the U-bar has a higher anchoring length and can better connect the wet joint to the prefabricated board. Additionally, in terms of failure deflection, the difference between the U-bar specimen (62.3 mm) and the hooked bar specimen (66.9 mm) is relatively small, at only 7.4%, but this value is much larger than that of the headed bar specimen (16.2 mm) and the linear bar specimens (13.4 mm and 26.0 mm, respectively). U-bar and hooked bar wet joints exhibit superior ductility, whereas headed bar and linear bar wet joints demonstrate inferior ductility. Despite the high bearing capacity of headed bar wet joints, their inadequate ductility necessitates cautious or even avoided use in engineering practice. A comparative analysis of the mechanical properties of lapped linear bar specimens and welded linear bar specimens reveals that welding has limited enhancement effects on the performance of linear bar wet joints. Both types of specimens exhibit relatively low ultimate loads and failure deflections, thus warranting careful consideration or avoidance of linear bar wet joints in engineering applications.

**Figure 5:**
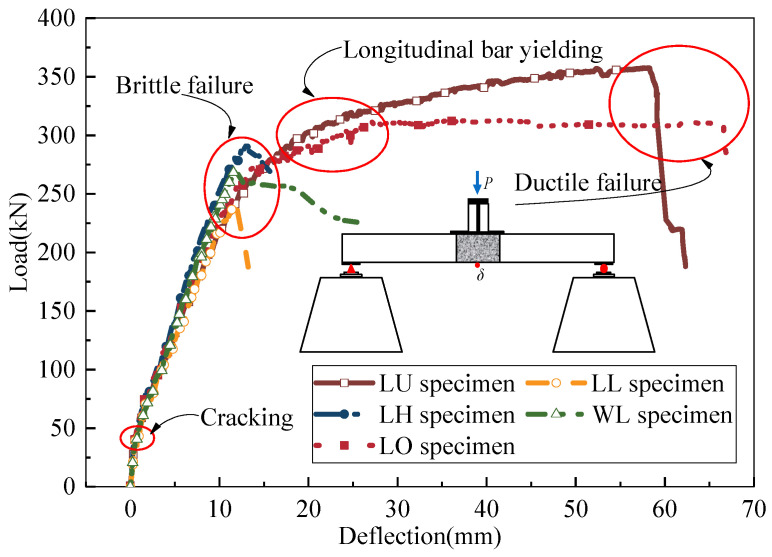
Load–deflection curves.

Figure 5 illustrates the load–deflection curves of the specimens. Upon observing the figure, it is evident that the load–deflection curves of U-bar and hooked bar specimens exhibit a pronounced “plateau stage”. During this stage, when the nominal yield load is reached, the longitudinal steel bars in the wet joint yield. Subsequently, the load increase becomes gradual while the deflection continues to grow, ultimately leading to specimen failure due to concrete crushing in the compression zone of the bridge deck. This process demonstrates a ductile failure mode, indicating that U-bars and hooked bars can provide sufficient anchorage capacity, thereby fully exerting the effectiveness of the longitudinal steel bars within the wet joint. Conversely, the load–deflection curves of the headed bar and linear bar specimens do not exhibit a “plateau stage”. After reaching the ultimate load, the load suddenly drops, and the specimens undergo brittle failure. Because the stress mode of the lapped-headed bars joint is the strut and tie model, the tie members (i.e., longitudinal steel bars) have not reached their yield stress while the compression strut (i.e., concrete) fails, so the specimen immediately suffers brittle failure. The anchorage length of the linear bars was not enough, which led to the longitudinal steel bars not yielding at the time of specimen failure, and thus, the effectiveness of the steel bars was not fully utilized.

Based on the aforementioned comparative analysis, we can draw the following conclusion: lapped U-bar wet joints exhibit superior performance in both bearing capacity and ductility, providing adequate anchorage capacity and fully exerting the role of longitudinal steel bars prior to specimen failure. Compared to lapped hooked, headed, and linear bar wet joints, lapped U-bar wet joints demonstrate more advantageous mechanical properties.

### 3.2. Crack Mode

**Figure 6:**
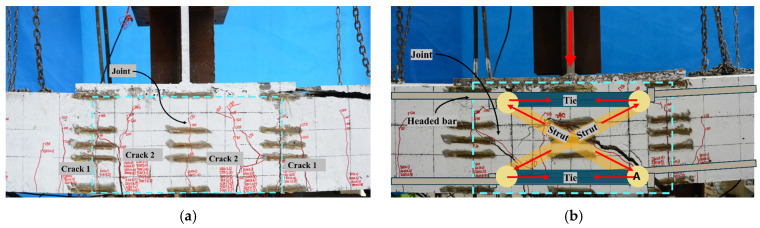

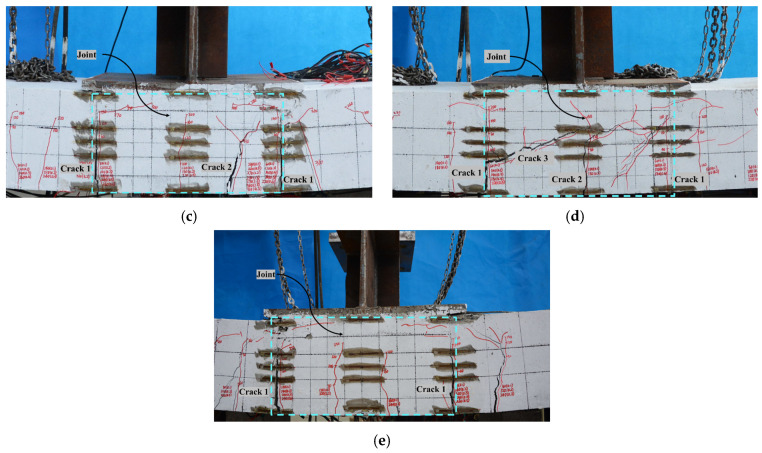
Crack patterns of five joint specimens. (**a**) LU; (**b**) LH; (**c**) LO; (**d**) LL; (**e**) WL.

U-shaped and hooked bar wet joints exhibit similar crack patterns, as shown in Figure 6a,c. When the load reaches 40 kN, the first crack (Crack 1) appears at the interface between the new and old concrete. As the load continues to increase, the width of Crack 1 gradually widens, and new cracks continuously emerge. When the load reaches 220 kN, the second crack (Crack 2) appears within the wet joint, 100 mm away from the interface between the new and old concrete. As the load further increases, the width of Crack 2 continues to widen, while Crack 1 gradually closes. When the specimen fails, the width of Crack 2 reaches 4–5 mm. This phenomenon indicates that the strain of the longitudinal steel bars develops fully after yielding, and the role of the longitudinal steel bars is fully utilized. Upon concrete crushing in the compression zone, the specimen loses its bearing capacity and exhibits ductile failure characteristics.

The primary crack in headed bar wet joints during failure is a diagonal shear crack. When the load reaches 40 kN, the crack first appears at the interface between the new and old concrete. With the increase in load, the crack expands gradually, the width increases, and the length extends, showing an obvious diagonal line. The mechanical behavior of headed bar wet joints can be analyzed using the strut-and-tie model [14], as illustrated in Figure 6b. In this model, the headed bars serve as tie members, bearing tensile forces, while the concrete between the headed bars in the upper and lower layers acts as a compression strut, bearing compressive forces. Notably, the headed bars at the joint locations provide adequate anchorage capacity. If the tie members (i.e., longitudinal steel bars) have not reached their yield stress while the compression strut (i.e., concrete) fails, the failure mode shown in Figure 6b will occur. In this case, diagonal shear cracks will form between the headed bars in the upper and lower layers, and vertical force imbalance will arise at node A, causing significant downward displacement of the concrete at node A. Such failure mode of headed bar wet joints should be avoided in engineering practice. Therefore, during the design phase, it is imperative to ensure that the diagonal concrete (compression strut) does not fail before the longitudinal steel bars (tie members) yield.

For lapped linear bar specimens, when the load reaches 38 kN, the first crack (crack 1) appears at the interface between the new and old concrete. As the load continues to increase, the width of Crack 1 gradually widens, and new cracks continuously emerge. When the load reaches 80 kN, the second crack (Crack 2) appears within the wet joint, 250 mm away from the interface between the new and old concrete. As the load further increases, the lapped linear bar cannot provide sufficient anchorage capacity, leading to slip between the longitudinal steel bars and the concrete, which induces diagonal cracks (crack 3) within the wet joint (as shown in Figure 6d). In such cases, brittle failure of the specimen often occurs before the longitudinal steel bars yield. For welded linear bar specimens, When the load reaches 35 kN, the first crack (crack 1) appears at the interface between the new and old concrete. With the increase in load, the welded linear bar specimen will not produce diagonal cracks, which is different from the lapped linear bar specimen. This is because welding restricts the slip of straight steel bars, welded linear bar wet joints are prone to bending cracks (as shown in Figure 6e), with the primary crack typically located at the interface between new and old concrete. This indicates that welded linear bars also fail to provide adequate anchorage capacity.

### 3.3. Reinforcing Steel Strain

**Figure 7:**
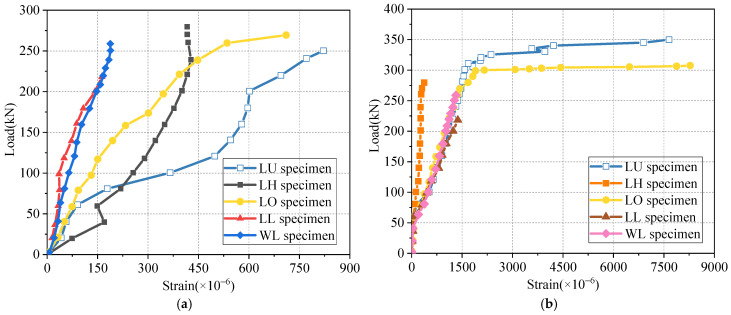
Load versus longitudinal reinforcement strain comparison. (**a**) End of reinforcing steel; (**b**) At a distance of 400 mm from the ends of bars.

To accurately measure the strain in the longitudinal steel bars, we use the average values of strain gauges 1-0 and 2-0 to represent the strain at the ends of the bars, while the strain at a distance of 400 mm from the ends is reflected by the average values of strain gauges 1–400 and 2–400 (as shown in Figure 4b). Figure 7 presents the relationship between the longitudinal steel bar strain and the applied load. According to Figure 7a, it can be seen that at the initial stage of load application, obvious strain appears at the end of the longitudinal steel bar. During the whole loading process, the steel bar is still in the elastic stage because the strain of the steel bar at the end of LU and LO specimens is less than 1000 με, and that of LH, LL, and WL is less than 500 με. In addition, it is worth noting that the end steel strain of the U-shaped bar lap specimen is larger than that of the other four specimens, which indicates that the wet joints of the U-shaped bar lap have stronger connection properties. According to Figure 7b, When the load reaches 40 kN, significant strain appears at a location 400 mm from the ends of the bars, which corresponds to the cracking load of the specimen. During the failure of lapped/welded linear bar wet joints, the strain in the longitudinal steel bars is less than 2000 με, indicating that the longitudinal steel bars have not yielded, Due to the insufficient anchorage length of longitudinal steel bars, the function of steel bars is not fully played, and resulting in brittle failure of the specimen. In contrast, when the load reaches 300 kN, the longitudinal steel bars in the U-bar and hooked bar wet joints yield, and the load-strain curve exhibits a plateau segment, indicating ductile failure of the specimen. Notably, the strain at a distance of 400 mm from the ends of headed bars remains relatively low, which further confirms that the mechanical behavior of headed bar wet joints differs significantly from that of the other four types of wet joints. This phenomenon also validates the rationality of the strut-and-tie model analysis presented in Section 2.2, where headed bars, acting as tie members, experience relatively small tensile forces.

**Figure 8:**
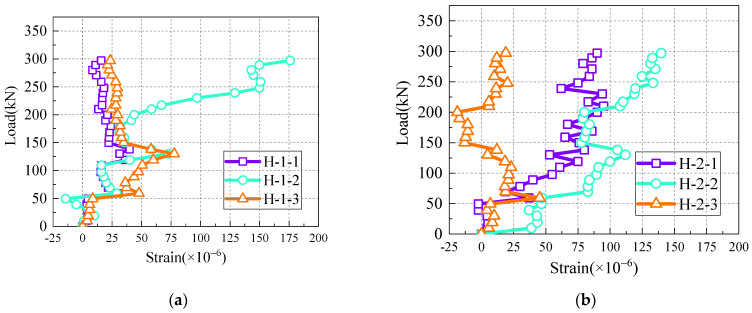
Load versus transverse reinforcement strain plot for the LU specimen. (**a**) The first transverse bar; (**b**) The second transverse bar.

During the experimental observations, the strain values of the transverse reinforcement within the core concrete of the wet joints consistently remained below 200 με, indicating that the forces borne by the transverse reinforcement in the wet joints were relatively small. Figure 8 illustrates the load-transverse reinforcement strain curve for U-bar wet joints. Notably, the load-transverse reinforcement strain curves for wet joints with headed bar, hooked bar, and linear bar exhibited similar trends to those of the U-bar wet joints.

## 4. Finite Element Model

Through a series of experimental validations, U-bar wet joints have demonstrated superior mechanical properties, characterized by their highest load-bearing capacity and good ductility. To gain a deeper understanding of their mechanical characteristics, this study employed ABAQUS 2022 to successfully establish a nonlinear finite element model for U-bar wet joint specimens. By comparing the finite element analysis results with experimental data, it was confirmed that the model could accurately simulate the mechanical properties of U-bar wet joints. Furthermore, using this finite element model, the study simulated the specific effects of lap length (H) and axial compressive strength of wet joint concrete (*f*_ck_) on the mechanical properties of U-bar wet joints.

### 4.1. Finite Element Models and Meshing

**Figure 9:**
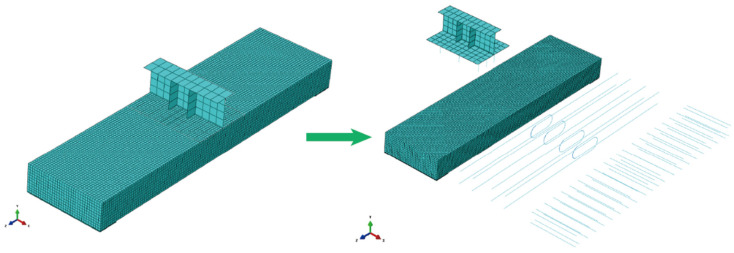
Non-linear finite element model of the LU Specimen.

During the simulation process, the concrete bridge deck was modeled using C3D20R solid elements, while the I-shaped steel beams were simulated using S4R shell elements. The longitudinal reinforcement, transverse reinforcement, and studs were uniformly modeled using T3D2 truss elements. To ensure the authenticity of the simulation, the reinforcement and studs were embedded into the concrete bridge deck. At the interface between the I-shaped steel beams and the concrete bridge deck, normal hard contact was set up, and the tangential friction coefficient was set to 0.3. Additionally, the studs were bonded to the top plate of the I-shaped steel beams. Taking into account both computational efficiency and precision, a mesh size of 25 mm was ultimately chosen for the concrete. In a similar vein, a mesh size of 50 mm was assigned to both the longitudinal and transverse reinforcements. As bearings and loading beams are not the primary focus of the analysis, they were modeled as rigid bodies with a mesh size of 100 mm. Figure 9. clearly presents the nonlinear finite element model of the U-shaped steel bar specimen.

### 4.2. Boundary Conditions and Loading

To establish the simply supported boundary conditions for the specimen, two base plates were set at both ends of the specimen. Specifically, the centerline of the left base plate was constrained in terms of displacement in the X, Y, and Z directions, while the centerline of the right base plate was constrained in the X and Y directions. Next, a reference point was set at the center of the bottom plate of the I-shaped steel beam, and the degrees of freedom of this reference point were coupled with those of the bottom surface of the I-shaped steel beam. Finally, displacement loading was applied to this reference point along the negative Y-axis direction to complete the entire loading process.

### 4.3. Material Constitutive Models

**Figure 10:**
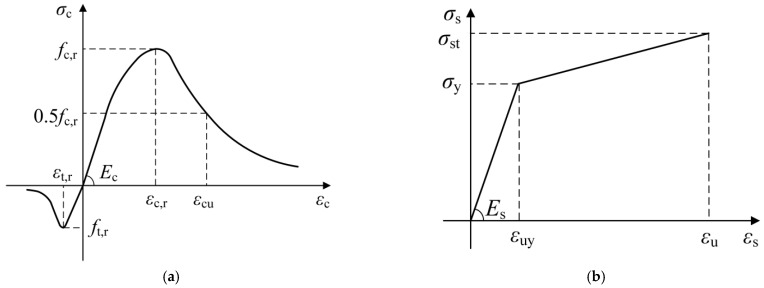
Material constitution of concrete and steel. (**a**) Concrete; (**b**) Steel.

To simulate the complex nonlinear behavior of concrete, this study employed a damage plasticity model. In constructing the constitutive model for concrete, the relevant provisions of the standard GB 50010 [30] were strictly followed, as shown in Figure 10a. Meanwhile, the parameters of the concrete damage plasticity model are detailed in Table 2. For the constitutive model of steel reinforcement, the linear reinforcement model specified in the standard GB 50010 [30] was adopted, as shown in Figure 10b. As for the studs and I-shaped steel beams, an ideal elastoplastic model was used for simulation. The constitutive parameters of steel reinforcement and steel materials can be found in Table 3.

### 4.4. FEM Results

**Figure 11:**
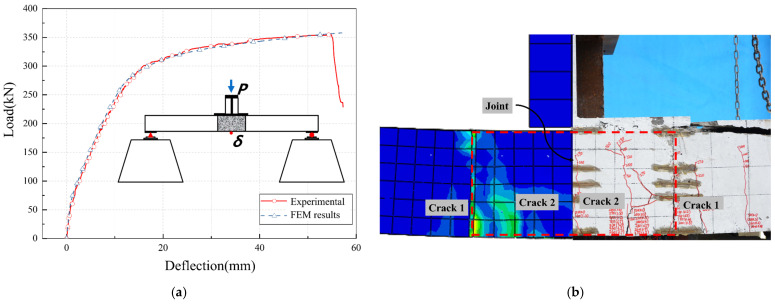
Comparison between the FEM and experimental results. (**a**) Comparison of load–deflection curves; (**b**) Comparison of crack patterns.

Figure 11 presents a comparison of the load–deflection curves and crack patterns obtained from the finite element model calculations with the experimental results. It is worth noting that the descending segment of the load–deflection curve is challenging to model accurately due to brittle failure, which is an inherent limitation of finite element (FE) analysis. Nonetheless, FE results demonstrate a high degree of consistency with experimental findings, indicating their accurate capability in predicting the deformation process. As shown in Figure 11a, the bearing capacity calculated by the finite element method is 351.3 kN, which is only 1.8% lower than the experimental result (357.6 kN). This comparison demonstrates that the finite element model can accurately simulate the load–deflection relationship and crack patterns of the lapped U-bar wet joint specimen. As shown in Figure 11b, The finite element cracking mode is also highly consistent with the experimental results. Cracks first appear at the interface, and then, with the increase of load, bending cracks appear at the bottom of the wet joint.

### 4.5. Revised Formula

**Figure 12:**
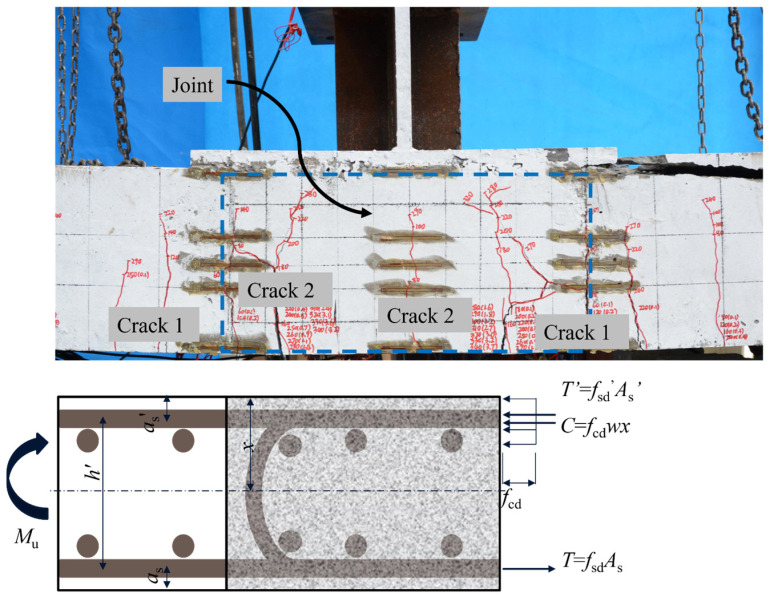
Calculation of the bending capacity of the LU specimen.

Based on the experimental data and finite element analysis results, we can conclude that the mechanical behavior and failure process of the U-bar wet joint specimen are similar to those of double-reinforced beams. In the U-bar wet joint specimen, the bottom steel reinforcement is in tension, while the top steel reinforcement and the concrete in the compression zone bear pressure. The tensile force in the bottom steel reinforcement balances the compressive forces in the concrete and steel in the compression zone. Simultaneously, the top steel reinforcement, together with the concrete and compressed steel in the compression zone, forms a moment to counteract the external moment. To visually demonstrate this, a bearing capacity calculation model for the U-bar wet joint specimen has been established, as shown in Figure 12.

From the equilibrium condition that the sum of internal forces in the horizontal direction of the section is zero, i.e., *T* + *C* + *T*’ = 0, we can derive

(1) $$f_{\mathrm{cd}}wx+f_{\mathrm{sd}}^{\prime }A_{s}^{\prime }=f_{\mathrm{sd}}A_{s}$$

In the formula, *T* represents the resultant tensile force in the reinforcing steel, *C* represents the resultant compressive force in the concrete, *T*’ represents the resultant compressive force in the compressed reinforcing steel, $f_{\mathrm{cd}}$ denotes the axial compressive strength of the concrete, *w* is the width of the section, *x* is the height of the compression zone calculated using the equivalent rectangular stress block method, $f_{\mathrm{sd}}^{\prime }$ is the compressive strength of the reinforcing steel in the compression zone, $A_{s}^{\prime }$ is the cross-sectional area of the reinforcing steel in the compression zone, $f_{\mathrm{sd}}$ is the tensile strength of the reinforcing steel in the tension zone, $A_{s}$ is the cross-sectional area of the reinforcing steel in the tension zone.

From the equilibrium condition that the sum of moments about the point of application of the resultant tensile force *T* in the section is zero, we can derive

(2) $$M_{u}=f_{\mathrm{cd}}wx\left(h_{0}-x/2\right)+f_{\mathrm{sd}}^{\prime }A_{s}^{\prime }\left(h_{0}-a_{s}^{\prime }\right)$$

In the formula, $M_{u}$ represents the bending capacity of the section, $h_{0}$ is the effective depth of the section, $a_{s}^{\prime }$ is the distance from the resultant force point of the compressed reinforcing steel to the compressed edge of the section.

Based on Equation (1), we obtain *x* = 0. According to the standard JTG D60-2015 [34], in this case, *x* can be taken as −2$a_{s}^{\prime }$, assuming that the point of action of the resultant compressive stress in the concrete coincides with the point of action of the resultant force of the compressed reinforcing steel $A_{s}^{\prime }$ (Figure 12). The approximate expression for the bending capacity of the section is then given by

(3) $$M_{u}=f_{\mathrm{sd}}A_{s}\left(h_{0}-a_{s}^{\prime }\right)$$

Compared to conventional double-reinforced beam sections, the longitudinal reinforcement in U-bar wet joints is not continuously arranged but is instead lap using U-bars. If the lap length is insufficient, the wet joint may fail due to the sliding effect of the U-bars. If the strength of the wet joint concrete is inadequate, the joint may fail due to shear failure of the core concrete. Therefore, the bearing capacity of U-bar wet joint specimens is significantly influenced by both the lap length (H) and the axial compressive strength *f*_ck_ of the wet joint concrete.

**Figure 13:**
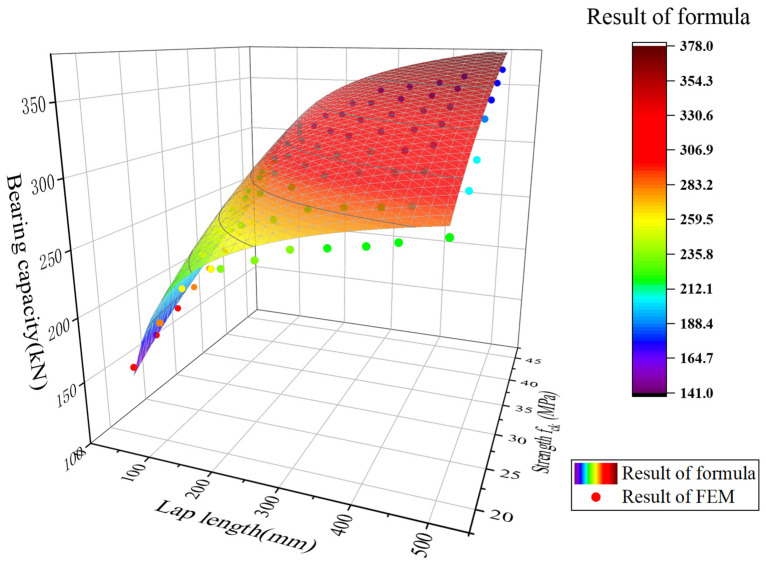
Comparison of the formula and FEM results.

In this study, a finite element method was employed to conduct a parametric analysis of the lap length (H) and the axial compressive strength *f*_ck_ of wet joint concrete. Specifically, the lap length was set to vary from 50 mm to 500 mm, with 10 groups at intervals of 50 mm. The axial compressive strength of the wet joint concrete ranged from 13.4 MPa to 41.5 MPa, encompassing 8 strength grades (corresponding to concrete grades from C30 to C65). By conducting a coupled analysis of these two parameters, a total of 80 nonlinear finite element models were constructed and computed. The results of the computations are presented as scatter plots in Figure 13. The bearing capacity of lapped U-bars joints gradually increases with the extension of lap length and the enhancement of concrete strength. However, when the lap length exceeds 100 mm, the rate of increase in load-bearing capacity tends to decline.

Based on the bending capacity calculation formula for double-reinforced beam sections, this study introduces a self-organizing migration algorithm to deeply analyze the coupled effects of lap length and concrete strength. Through this method, a formula for calculating the bearing capacity of U-shaped steel bar wet joint specimens has been derived. This formula comprehensively considers several key factors, including the lap length H, the axial compressive strength *f*_ck_ of the wet joint concrete, the yield strength *f*_yL_ of the U-shaped steel bars, the cross-sectional area *A*_s_ of the U-shaped steel bars, the spacing h’ between the upper and lower layers of steel bars, and the calculated span *l*, all of which significantly influence the bearing capacity of the U-shaped steel bar wet joint specimens.

(4) $$P_{n}=4\alpha f_{\mathrm{yL}}A_{s}h^{\prime }/l$$

(5) $$\alpha =-3.9/\sqrt{H}+0.32\ln\left(f_{ck}\right),H\leq 500\;mm$$

A comparison between the bearing capacity of U-shaped steel bar wet joint specimens calculated using Equation (4) and the results of finite element analysis is presented in Figure 13. In the figure, the scattered points represent the results of the finite element analysis, while the surface represents the results calculated using Equation (4). By comparing the two, it is found that the average ratio of the results calculated using Equation (4) to those of the finite element analysis is 1.03, with a variance of only 0.0004, indicating a high degree of consistency between the two.

## 5. Conclusions

This study conducted bending-shear tests on five wet joint specimens; comparative analyses were conducted on the bearing capacity, failure modes, crack distribution, failure deflection, and steel bar strain of the specimens. Additionally, based on the test results and parameter analysis results, a formula for calculating the bearing capacity of lapped U-bar joints was proposed. The main conclusions are as follows:

1. In comparison to large-headed reinforcement, hooked reinforcement, and straight reinforcement, U-shaped reinforcement wet joints exhibit the highest bearing capacity, reaching 357.6 kN. This is a significant increase of 22.8%, 14.2%, 50.4%, and 32.1% over the bearing capacities of head bar wet joints (291.2 kN), hooked reinforcement wet joints (313.0 kN), straight reinforcement lap joints (237.7 kN), and welded wet joints (270.7 kN), respectively. This is because the U-bar has a higher anchoring length and can better connect the wet joint to the prefabricated board.
2. The failure deflection of U-shaped reinforcement and hooked reinforcement wet joints is 62.3 mm and 66.9 mm, respectively, indicating ductile failure, while the failure deflection of head bar and linear bar wet joints is 16.2 mm, 13.4 mm, and 26.0 mm, respectively, indicating brittle failure. Therefore, the lapped head bar and linear bar joint should be avoided as far as possible in the project.
3. In the specimens with lapped/welded linear bars, the longitudinal bar strains at both the end and at a distance of 400 mm from the end were less than 2000 με; this is attributed to insufficient anchorage length, leading to steel bar slip and an inability to effectively share loads with the concrete. For specimens with lapped-headed bars, the longitudinal steel bar strains were all less than 500 με, which is related to the mechanical behavior of the wet joints in such large-headed bars. In specimens with U-bars and hooked bars, the longitudinal steel bar strains at a distance of 400 mm from the end exceeded 7500 με, causing the steel bars to yield and demonstrating good ductility in the specimens. Across all specimens, the transverse bar strains were less than 200 με, indicating that the transverse bars remained in a fully elastic state.
4. Experimental and finite element studies have revealed that the failure process and mechanical behavior of U-shaped reinforcement wet joint specimens are similar to that of double-reinforced beams, and the bearing capacity of U-shaped reinforcement wet joints is also influenced by the lap length and the strength of the wet joint concrete. Parameter analysis was conducted on the specimens using nonlinear finite element methods, and a formula was fitted based on the calculation results using a self-organizing migrating algorithm. The average ratio of the formula calculation results to the finite element calculation results is 1.03, with a variance of 0.0004.
5. Experimental studies have found that the mechanical behavior of large-head reinforcement wet joints can be analyzed using a strut-and-tie model. More experiments and finite element analyses are needed in the future to further investigate the mechanical behavior of large-head reinforcement wet joints.
6. The steel consumption of lapped U-bars joint is slightly higher compared to the other four connection forms. This is due to the addition of a U-shaped hook to the reinforcement, which may increase steel usage by less than 0.5%. The construction process is more complex, as one end of the reinforcement needs to be bent into a U-shape.

## Figures and Tables

**Table 1:** Testing results of five joint types.

Specimen	Connection Type	$\mathrm{Cracking}\;\mathrm{Load}\;P_{y}$(kN)	$\mathrm{Ultimate}\;\mathrm{Load}\;P_{n}$(kN)	$\mathrm{Failure}\;\mathrm{Deflection}\;\delta_{s}$(mm)
LU	Lapped U-bar	40	357.6	62.3
LH	Lapped headed bar	40	291.2	16.2
LO	Lapped hooked bar	40	313.0	66.9
LL	Lapped straight bar	38	237.7	13.4
WL	welded straight bar	35	270.7	26.0

**Table 2:** ABAQUS concrete constitutivemodel parameters.

Parameters	Values
Young’s modulus	3.6 × 10^4^ MPa
Poisson’s Ratio	0.2
Dilatancy Angle	36
Eccentricity	0.1
Ratio of Initial Equibiaxial Compressive Yield Stress to Initial Uniaxial Compressive Yield Stress	1.16
Second Invariant of Stress on Tensile Meridional Plane	0.6667
Viscosity Coefficient	2.5 × 10^−5^
Axial Compressive Strength of Wet Joint Concrete	39.8 MPa
Axial Compressive Strength of Precast Concrete Panel	35.5 MPa

**Table 3:** Constitutive model parameters for the reinforcement and steel.

	Young’s Modulus (GPa)	Yield Stress (MPa)	Yield Strain	Ultimate Stress (MPa)	Ultimate Strain
Longitudinal Reinforcing Steel	195	460	0.002	603	0.16
Transverse Reinforcing Steel	195	507	0.002	615	0.16
I-shaped Steel Beams	210	345	-	-	-
Studs	210	345	-	-	-

## Data Availability

The original contributions presented in this study are included in the article. Further inquiries can be directed to the corresponding authors.
